# XM02 is superior to placebo and equivalent to Neupogen™ in reducing the duration of severe neutropenia and the incidence of febrile neutropenia in cycle 1 in breast cancer patients receiving docetaxel/doxorubicin chemotherapy

**DOI:** 10.1186/1471-2407-8-332

**Published:** 2008-11-12

**Authors:** A del Giglio, A Eniu, D Ganea-Motan, E Topuzov, H Lubenau

**Affiliations:** 1 Auro del Giglio, Faculdade de Medicina do ABC, Santo André, Sao Paulo, Brazil and Hospital Israelita Albert Einstein Sao Paulo, Brazil; 2 Alexandru Eniu, Institutul Oncologic Ion Chiricuţă, Cluj-Napoca, Romania; 3 Doina Ganea-Motan, Spitalul Judetean de Urgenta, Suceava, Romania; 4 Eskender Topuzov, Mechnikov State Medical Academy, Saint-Petersburg, Russia; 5 Heinz Lubenau, BioGeneriX AG, Mannheim, Germany

## Abstract

**Background:**

Recombinant granulocyte colony-stimulating factors (G-CSFs) such as Filgrastim are used to treat chemotherapy-induced neutropenia. We investigated a new G-CSF, XM02, and compared it to Neupogen™ after myelotoxic chemotherapy in breast cancer (BC) patients.

**Methods:**

A total of 348 patients with BC receiving docetaxel/doxorubicin chemotherapy were randomised to treatment with daily injections (subcutaneous 5 μg/kg/day) for at least 5 days and a maximum of 14 days in each cycle of XM02 (n = 140), Neupogen™ (n = 136) or placebo (n = 72). The primary endpoint was the duration of severe neutropenia (DSN) in cycle 1.

**Results:**

The mean DSN in cycle 1 was 1.1, 1.1, and 3.9 days in the XM02, Neupogen™, and placebo group, respectively. Superiority of XM02 over placebo and equivalence of XM02 with Neupogen™ could be demonstrated. Toxicities were similar between XM02 and Neupogen™.

**Conclusion:**

XM02 was superior to placebo and equivalent to Neupogen™ in reducing DSN after myelotoxic chemotherapy.

**Trial Registration:**

Current Controlled Trials ISRCTN02270769

## Introduction

Myelotoxic chemotherapy frequently leads to neutropenia. Recombinant granulocyte colony-stimulating factors (G-CSFs) are effective pharmaceutical substances and are successfully applied in the prevention of chemotherapy-induced neutropenia and the associated risk of infection. [1-3]

Natural human G-CSF is a glycoprotein composed of a single polypeptide chain of 174 or 177 amino acids.[4,5] The first bacterially synthesised non-glycosylated recombinant methionyl form of human G-CSF (r-metHuG -CSF) was approved by the Food and Drug Administration (FDA) in 1991 under the generic name Filgrastim. Alternatively, a second glycosylated recombinant human G-CSF is available on the market under the generic name Lenograstim which is produced by Chinese hamster ovary (CHO) cells.

More recently, a biosimilar non-glycosylated r-metHuG-CSF expressed in *Escherichia coli *for intravenous (i.v.) and subcutaneous (s.c.) administration was clinically developed by BioGeneriX AG for the treatment of chemotherapy-induced neutropenia. The manufacturing process was developed by Sicor Biotech. XM02 will be named with the international non-proprietary name filgrastim. Filgrastim marketed under the trade name Neupogen™ was used as reference product in this study.

The primary aim of the study was to demonstrate the activity of XM02 compared to Neupogen™ and placebo. We show that XM02 is safe and well tolerated in breast cancer patients undergoing chemotherapy and is very similar to Neupogen™ in effectively stimulating neutrophil recovery.

## Methods

### Patients

Between December 2004 and September 2005, patients with breast cancer requiring chemotherapy participated in this multinational, multicentre, randomised and controlled phase III study at 52 study centres in 10 countries. The study was approved by all local institutional review boards and ethics committees concerned. The first ethics committee approval was given by the National Ethics Committee of Medicamentului Student Clinic, Bucharest, subsequently followed by the approvals for all 52 centres involved. Male and female patients ≥ 18 years of age with breast cancer high risk stage II, III or IV (classification according to American Joint Committee on Cancer) were eligible to participate if they signed written informed consent, were planned/eligible to receive treatment with docetaxel/doxorubicin as routine chemotherapy for their breast cancer, were chemotherapy-naïve, had Eastern Cooperative Oncology Group performance status ≤ 2, an absolute neutrophil count (ANC) ≥ 1.5 × 10^9^/L, platelet count ≥ 100 × 10^9^/L, adequate cardiac function (including left ventricular ejection fraction ≥ 50% as assessed by echocardiography within 4 weeks prior to randomisation), adequate hepatic function, i.e., alanine and aspartate aminotransferases <2.5 × upper limit of normal (ULN), alkaline phosphatase <5 × ULN, bilirubin <ULN, and adequate renal function, i.e., creatinine <1.5 × ULN.

### Methods

A total of 348 patients were randomised in a 2:2:1 ratio to treatment with either XM02 (n = 140), Neupogen™ (n = 136) or placebo (n = 72). Patients in the placebo group switched to treatment with XM02 after completion of cycle 1. Patients underwent a maximum of 4 chemotherapy cycles (3 weeks per cycle), consisting of doxorubicin 60 mg/m^2 ^(i.v. bolus injection) and docetaxel 75 mg/m^2 ^(at least 1 hour infusion). Starting one day after chemotherapy, patients received daily injections of either XM02 or Neupogen™ (both s.c. 5 μg/kg/day based on actual body weight) or s.c. placebo (in the first cycle only) for at least 5 days and a maximum of 14 days. The dose of XM02 was chosen based on bioequivalence between XM02 and Neupogen™ previously demonstrated in healthy volunteers [6]. Both used G-CSF had to be stopped when an ANC of ≥ 10 × 10^9^/L after nadir was reached. Blood samples for the determination of the ANC were taken within 24 hours before chemotherapy and then daily from Day 2 until Day 15, or longer until ANC reached ≥ 2.0 × 10^9^/L. Body temperature (axillary) was measured with a standardised device daily until Day 15, or longer until ANC reached ≥ 2.0 × 10^9^/L.

A true double-blind design was not feasible because XM02 and Neupogen™ were formulated in different volumes. For this reason, the study drug was administered by qualified unblinded study personnel and the investigator was kept blinded and performed all assessments of the patient without knowledge of treatment. Moreover, it was considered that the efficacy endpoints of this study (based on ANC) were very unlikely to be influenced by the investigator's or patient's knowledge of the treatment. During cycle 1, safety was closely monitored by an independent, unblinded Safety Monitoring Board in order to ensure that patients in the placebo arm were not exposed to an unjustifiable risk.

### Endpoints and definitions

The primary endpoint was the duration of severe neutropenia (DSN) in cycle 1, defined as the number of days with grade 4 neutropenia with an ANC <0.5 × 10^9^/L. Secondary endpoints included incidence of observed febrile neutropenia (FN) (observed FN defined as body temperature of >38.5°C for more than 1 hour and ANC <0.5 × 10^9^/L, both measured on the same day) and of protocol defined FN (administration of systemic antibiotics) by cycle and across all cycles, DSN in cycles 2 to 4, depth of ANC nadir in cycles 1 to 4, and time to ANC recovery in cycles 1 to 4. Safety assessments were based on adverse events (AEs), safety laboratory at the beginning of each cycle and at the end of study, immunogenicity samples before treatment, after the end of each cycle and 180 days after the start of treatment, physical examinations, and vital signs. In a subgroup of patients per treatment group pharmacokinetic samples were taken in cycle 1 and cycle 4 on day 1 and after repeated dosing on the day after the ANC had reached 2 × 10^9^/L. Serum concentrations were determined with the enzyme-linked immunosorbent assay test (ELISA) kit (Quantikine^®^, R&D Systems, USA) by Cirion Biopharma Research Inc., Canada.

### Statistical methods

First, assay sensitivity with respect to DSN in cycle 1 was confirmed by comparing XM02 versus placebo. If this difference was significant (analysis of covariance [ANCOVA], two sided p ≤ 0.05 with shorter DSN for XM02), equivalence between XM02 and Neupogen™ was assessed. To show equivalence between XM02 and Neupogen™, the 2-sided 95% confidence interval (CI) for the difference in DSN in cycle 1 had to lie entirely within the equivalence range of [-1 day, +1 day]. A difference of 1 day was considered to be the maximum clinically acceptable difference. Safety endpoints were summarised using descriptive statistics and the incidence of AEs was compared for XM02 versus Neupogen™ using Fisher's exact test.

## Results

The treatment groups were similar with regard to demographic and baseline characteristics. There were no relevant differences between treatment groups for incidence of prior or concomitant medications. Demographic and baseline characteristics are summarised in Table 1.

**Table 1:** Patient Characteristics

	**XM02** **(n = 140)**	**Neupogen™** **(n = 136)**	Placebo/XM02* (n = 72)
**Gender [N (%)]**			

Male	1 (0.7%)	1 (0.7%)	-
Female	139 (99.3%)	135 (99.3%)	72 (100.0%)

**Age [years]**			

Mean	51.0	51.4	49.5
SD	9.7	10.7	10.3
Median	51.0	51.0	48.0
Range	25–75	28–74	28–74

**Race [N (%)]**			

Caucasian	120 (85.7%)	118 (86.8%)	62 (86.1%)
Black	1 (0.7%)	5 (3.7%)	2 (2.8%)
Hispanic	10 (7.1%)	10 (7.4%)	6 (8.3%)
Other	9 (6.4%)	3 (2.2%)	2 (2.8%)

**Body Mass Index [kg/m^2^]**			

Mean	27.77	28.20	27.42
SD	6.11	5.70	6.02
Median	27.55	26.90	27.30
Range	16.2–56.2	15.9–45.4	17.0–41.3

**Cancer stage [N (%)]**			

High risk stage II	23 (16.4%)	36 (26.5%)	15 (20.8%)
Stage III	79 (56.4%)	69 (50.7%)	38 (52.8%)
Stage IV	38 (27.1%)	31 (22.8%)	19 (26.4%)

**Therapy [N (%)]**			

Adjuvant	96 (68.6%)	96 (70.6%)	47 (65.3%)
Metastatic	44 (31.4%)	40 (29.4%)	25 (34.7%)

**Prior radiation therapy [N (%)]**			

No	125 (89.3%)	127 (93.4%)	63 (87.5%)
Yes	15 (10.7%)	9 (6.6%)	9 (12.5%)

Abbreviations: SD = Standard deviation,

Body Mass Index calculated as body weight/(height)^2^

*Patients in this group received placebo in cycle 1 and XM02 afterwards

### Efficacy

The patients were exposed to the study drug for a median of 38 days (range: 1 to 55 days). Median duration within a cycle was 9 or 10 days (range 1 to 16 days). The patients were exposed to a mean of 15,739.6 μg (range: 540 to 29,280 μg) of active study drug (XM02 or Neupogen™). There were no differences between the XM02 and Neupogen™ groups with regard to amount of active study drug and the duration of exposure.

Results are summarised in Table 2 for the full analysis (FA) set.

**Table 2:** Results of Efficacy Endpoints

**Treatment group**	**XM02**	**Neupogen**™	Placebo/XM02*
**Full analysis set [n]**	**(n = 140)**	**(n = 136)**	**72**
**Mean DSN [days]**			
Cycle 1	1.1	1.1	3.8
ANCOVA [CI]#	0.028 [-0.261, 0.316]		
Cycle 4	0.7	0.7	0.6

**Mean ANC nadir [10^9^/L]**			
Cycle 1	0.7	0.7	0.2
ANCOVA [CI]#	-0.001 [-0.190, 0.189]		
Cycle 4	1.0	1.0	1.1

**Mean time to ANC recovery [days]**			
Cycle 1	8.0	7.8	14.0
ANCOVA [CI]#	0.207 [-0.425, 0.838]		
Cycle 4	7.6	7.1	7.2

**Incidence of FN [%]+**			
Cycle 1	12.1	12.5	36.1
Across all cycles	20.7	22.1	41.7

Abbreviations: DSN: duration of severe neutropenia; ANC: absolute neutrophil count; FN: febrile neutropenia; ANCOVA: analysis of covariance, CI: confidence interval.

* Patients in this group received placebo in cycle 1 and XM02 afterwards (including in cycle 4).

# ANCOVA estimate and 2-sided 95% confidence interval for difference XM02 – Filgrastim in cycle 1.

+ Observed or protocol defined FN.

#### Duration of Severe Neutropenia

In the per protocol (PP) set, mean DSN in cycle 1 was 1.1, 1.1, and 3.9 days in the XM02, Neupogen™, and placebo group, respectively. DSN ranged from 0 to 5 days in the XM02 and Neupogen™ groups, and from 0 to 9 days in the placebo group. Results were similar in the FA set, i.e., mean DSN in cycle 1 was 1.1, 1.1, and 3.8 days in the XM02, Neupogen™, and placebo group, respectively.

Superiority versus placebo and assay sensitivity were evaluated by comparing XM02 with placebo for the FA set. The least square mean of DSN was significantly shorter in the XM02 group (1.141 days) than in the placebo group (3.823 days). Thus, assay sensitivity was demonstrated. Results for the PP set were similar and confirmed superiority of XM02 over placebo and assay sensitivity.

Equivalence of XM02 and Neupogen™ was assessed based on the PP set, using the ANCOVA model to calculate a 2-sided 95% CI for "XM02 minus Neupogen™". The least square mean of DSN in cycle 1 was 1.119 and 1.087 days in the XM02 and Neupogen™ group, respectively. The 95% CI for "XM02 versus Neupogen™" was [-0.262 days, 0.325 days], which was entirely included in the pre-specified equivalence range [-1, 1], thus, equivalence was concluded. Results for the FA set were similar and confirmed equivalence of XM02 and Neupogen™.

The mean DSN in cycles 2 to 4 was similar in all treatment groups. Mean DSN was 0.7, 0.7, and 0.5 days in cycle 2, 0.6, 0.7, and 0.6 days in cycle 3, and 0.7, 0.7, and 0.6 days in cycle 4 in the XM02, Neupogen™, and placebo/XM02 group (treated with XM02 in cycles 2 to 4), respectively.

#### Febrile Neutropenia

In cycle 1, the incidence of observed or protocol defined FN was distinctly lower in the XM02 and Neupogen™ groups (12.1% and 12.5%, respectively) compared to the placebo group (36.1%). There were no significant differences with regard to FN incidence between the XM02 and Neupogen™ groups neither in cycle 1 nor across all cycles.

#### Absolute Neutrophil Count

**Figure 1:**
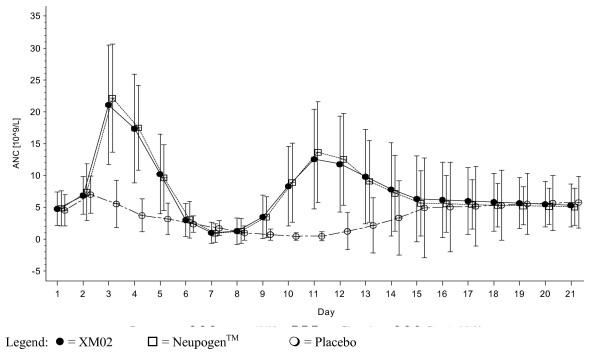
Mean (± SD) of Absolute Neutrophil Counts in Cycle 1 – FA Set.

In cycle 1 in the placebo group, mean ANC values decreased after Day 2 and reached a nadir on Day 11, whereas in the XM02 and Neupogen™ groups, mean values distinctly increased, reaching a maximum on Day 3, and then decreased to a nadir on Day 7. Thereafter, mean values in the active treatment groups distinctly increased again, reaching a maximum on Day 11. On Day 21, mean values returned to values as observed on Day 1 in all treatment groups. In the subsequent cycles, all treatment groups demonstrated the same trends as for XM02 and Neupogen™ in cycle 1 (see Figure 1).

In cycle 1, the mean ANC nadir was deeper in the placebo group (0.2 × 10^9^/L) compared to the XM02 and Neupogen™ groups (0.7 × 10^9^/L). In cycles 2, 3, and 4, the mean ANC nadir was not as deep as in cycle 1 and was similar across treatment groups with a mean value of approximately 1.0 × 10^9^/L.

In cycle 1, the median time to ANC recovery was shorter in the XM02 and Neupogen™ groups (8.0 and 8.0 days) compared to the placebo group (15.0 days). In cycles 2, 3, and 4, the time to ANC recovery was similar in all treatment groups with a median of 8.0 days.

### Adverse events

During the course of the study, 329 (94.5%) patients experienced a total of 3,268 AEs. Of these, 177 were considered as severe in 104 (29.9%) patients. There were 72 serious adverse events (SAEs) in 49 (14.1%) patients. Nine patients (2.6%) discontinued the study due to an AE, i.e., 2 (1.4%) patients in the XM02 group, 3 (2.2%) in the Neupogen™ group, and 4 (5.6%) in the placebo/XM02 group. The AEs leading to discontinuation in these nine patients were: sepsis, cardio-respiratory arrest, ischaemic stroke, syncope, pulmonary infarction, ALT increased, hyperglycaemia and myalgia, ALT and AST increased, thrombocytopenia. There were 3 deaths during the study treatment period (sepsis in the placebo group/cycle 1, cardiorespiratory arrest in the placebo group/cycle 1, and ischaemic stroke in the XM02 group/cycle 1), and 1 death after the end of study visit (metastasis in brain). All deaths were considered not related to the study drug.

The most commonly reported AEs were nausea (in 49.4% of patients), alopecia (48.0%), and asthenia (36.5%). Most commonly reported drug-related AEs included bone pain (10.3%), asthenia (7.8%), myalgia (6.3%), and diarrhoea (5.2%). In general, drug-related AEs occurred early in the study, i.e., they were reported within 15 days after study start and within the first 4 days of a cycle.

The AE profile was similar between the XM02 and Neupogen™ groups with exception of the incidence of drug-related AEs across all cycles, which were seen more frequently in the Neupogen™ group (in 39.7% of patients) than in the XM02 group (25.7%) (p = 0.0149).

Patients in the placebo group in cycle 1 had a higher incidence of AEs, SAEs, and severe SAEs compared to the XM02 and Neupogen™ groups. The Safety Monitoring Board decided to continue with the placebo group after 200 of a total of 350 planned patients had completed cycle 1.

Immunogenicity was low in all treatment groups. Few patients developed binding anti-G-CSF antibodies in all treatment groups whereas no confirmed plausible neutralising antibodies were detected.

### Pharmacokinetics

The pharmacokinetic profiles of XM02 and Neupogen™ were similar and t_1/2 _values correspond to published data on Neupogen™. In cycle 1 and cycle 4 in both profiles, mean serum concentrations of XM02 and Neupogen™ increased, reaching a maximum at 4 to 6 hours after dosing, and returned to pre-dose values by 24 hours. The median of t_1/2 _was similar in the XM02 and Filgrastim groups, i.e., 3.040 and 3.225 hours, respectively, in cycle 1 first profile, and 3.390 and 3.085 hours, respectively, in the second profile. In cycle 4, in both profiles, the median of t_1/2 _was similar in all treatment groups, ranging from 3.395 to 3.865 hours.

## Discussion

The primary aim of this study was to compare XM02 with placebo and Neupogen™ in terms of efficacy and safety in the treatment of chemotherapy-induced neutropenia. By testing of superiority of XM02 versus placebo, assay sensitivity with respect to the primary endpoint, DSN in cycle 1, was confirmed.

In this study, mean DSN in cycle 1 was 1.1 days for patients treated with Neupogen™ or XM02, and 3.9 days for patients receiving placebo. In the subsequent cycles, where all patients received XM02 or Neupogen™, mean DSN ranged from 0.5 to 0.7 days. These are similar results as observed in a study conducted by Holmes et al. [1], comparing filgrastim with pegfilgrastim in breast cancer patients, where mean DSN in the filgrastim group was 1.8 days in cycle 1, and ranged between 1.1 and 1.3 days in the subsequent cycles. The shorter DSN observed in the present study may be explained by the fact that only chemotherapy-naïve patients were included.

The incidence of observed or protocol defined FN in cycle 1 in the XM02 and Neupogen™ groups (12.1% and 12.5%, respectively) was about one third (33.5% and 34.6% respectively) compared to the placebo group (36.1%). In a study conducted by Timmer-Bonte et al. [7], the incidence of FN in cycle 1 during chemotherapy for lung cancer was 10% in patients treated with antibiotics plus G-CSF. The incidence of observed or protocol defined FN across all cycles in our study was 20.7% and 22.1% in the XM02 and Neupogen™ groups, respectively. Similar results were seen in the Holmes study [1], where 18% of patients in the Neupogen™ group developed FN across all cycles. This comparison has to be interpreted with caution as in the Holmes study [1] a different definition of FN was used. Brain et al. [8] reported an incidence for FN of 40.8% in a breast cancer study using doxorubicin 50 mg/m^2^/docetaxel 75 mg/m^2 ^and suggested to use primary G-CSF prophylaxis for this regimen. Vogel et al. [9] showed that pegfilgrastim markedly reduced the incidence of FN in breast cancer patients under moderate myelosuppressive chemotherapy with incidences of 1% with pegfilgrastim compared to 17% with placebo.

For patients receiving XM02 or Neupogen™, the ANC values distinctly increased after start of treatment, reaching a maximum on Day 3, and then decreased to a nadir on Day 7. Thereafter, ANC values increased again, reaching a maximum on Day 11. On Day 21, mean values returned to values as observed on Day 1. In a study conducted by Crawford et al. in patients with lung cancer under chemotherapy [10], in the filgrastim group, ANC values reached a maximum around Day 5, decreased to a nadir around Day 10 and reached a second maximum on Day 15. Holmes et al. [1] reported the same biphasic ANC profile under treatment with filgrastim in breast cancer patients treated with doxorubicin/docetaxel chemotherapy. In their study, the mean time to ANC recovery was 9.7 days for filgrastim, in our study the median time was 8.0 days for both XM02 and Neupogen™.

In general, the safety profile of XM02 and Neupogen™ was similar, whereas patients receiving placebo had distinctly more AEs and SAEs. Most commonly reported drug-related AEs were bone pain, asthenia, and myalgia, AEs that were expected from previous studies and experience with Neupogen™. In this study, drug-related AEs were seen significantly more frequently in the Neupogen™ than in the XM02 group.

In our study both G-CSF treatments were started one day after chemotherapy according to the Summary of Product Characteristics of Neupogen™ in order to properly compare both treatments. In clinical practice, G-CSF can also be administered starting from day 5 to 6 of each cycle [11]. This remains to be investigated in future studies with XM02 as well. More recently, pegfilgrastim, a pegylated form of filgrastim with a longer half life and duration of action was introduced into the market allowing a once per cycle administration [1].

In summary, XM02 treatment of breast cancer patients under docetaxel/doxorubicin resulted in a significant reduction of the DSN and the incidence of FN in cycle 1 to one third when compared to placebo. The reduction was similar in the XM02 and Neupogen™ group.

## Conclusion

In conclusion, treatment with XM02 is beneficial in ameliorating severe neutropenia and FN in breast cancer patients receiving myelosuppressive chemotherapy. XM02 is safe and well tolerated in the doses applied in this study.

## Conflicts of interests

This study was sponsored and funded by BioGeneriX AG.

A. del Giglio has a consultancy agreement with BioGeneriX AG. H. Lubenau is an employee of BioGeneriX AG.

## Authors' contributions

AG was coordinating investigator of the trial and participated in the design, conduct and evaluation of the study as well as in the manuscript writing. AE, DG, ET were involved in the conduct of the study and in the writing and review of the manuscript. HL was involved in the design, implementation, coordination and evaluation of the trial and in the drafting of the manuscript.

## Pre-publication history

The pre-publication history for this paper can be accessed here:


